# Are care workers appropriate mentors for nursing students in residential aged care?

**DOI:** 10.1186/s12912-014-0044-8

**Published:** 2014-12-12

**Authors:** Michael Annear, Emma Lea, Andrew Robinson

**Affiliations:** Wicking Dementia Research and Education Centre, University of Tasmania, Private Bag 143, Hobart, 7001 Tasmania Australia; School of Health Sciences and Wicking Dementia Research and Education Centre, University of Tasmania, Private Bag 143, Hobart, 7001 Tasmania Australia

**Keywords:** Aged care, Nursing, Clinical placement, Carer, Hygiene, Action research

## Abstract

**Background:**

The aged care sector is increasingly dominated by a less-qualified workforce at a time of increasing prevalence of complex health concerns, such as dementia. An Australian program to develop teaching aged care facilities is being undertaken to build the sector’s capacity and provide nursing students with positive experiences of engaging with vulnerable clients. This research aimed to examine care staff potential to facilitate nursing student engagement with clinically relevant knowledge in the performance of hygiene care in a residential aged care facility.

**Methods:**

This study was designed as an action research study. A cycle of reflection, planning, action, and evaluation is described to illustrate the carer mentor capacity to engage with and contribute to the learning of nursing students. Participants were second year student nurses (*n* = 10) on a four-week placement in a Tasmanian aged care facility in 2013 and their nurse/carer mentors (*n* = 17). Mentors participated in six action research meetings, and nursing students engaged in a parallel series of four feedback meetings during the placement.

**Results:**

At the beginning of the placement, nursing students exhibited a disregard for the clinical value of care provision. Students considered provision of hygiene care, in particular, the preserve of care workers and an inappropriate training exercise in the context of an undergraduate nursing qualification. To assist students to make links between core nursing competencies and hygiene care as well as to engender respect for their role within the aged care facility, carer mentors developed the Carer Assessment and Reporting Guide. Once implemented during the final weeks of the placement, the Guide improved student perceptions of resident hygiene care (reframed as assessment) and the role of facility care workers, as well as reinforcing carer self-esteem.

**Conclusion:**

Hygiene care is replete with nursing competencies that are valuable for undergraduate learners, including assessments of skin integrity, mobility, cognitive function, bowels and urine, and basic hygiene. Nurse education programs should strive to address student misconceptions about care work in facilities to account for population level increases in care needs.

## Background

In many developed countries, aged care has become increasingly dominated by a less-qualified workforce. In Australia, the number of care workers (also known as nurse aides or assistants) employed in residential aged care facilities (RACFs) or nursing homes rose 10% in a decade to comprise 68% of the total workforce [1]. Simultaneously, the proportion of qualified nurses in the sector declined from 34% to 27% [1]. Data also suggest that care workers comprise a large and growing sector of the United Kingdom workforce [2]. Such developments are problematic when considering RACF residents’ changing demographic composition, where those admitted have a higher dependence level and are more likely to have dementia [3]. In this environment, recruitment and retention of skilled nursing staff to meet residents’ growing needs has become difficult [4,5]. To redress a widening gap between workforce quality and demand for care, the Wicking Dementia Research and Education Centre (University of Tasmania) has instituted a program to develop a network of Teaching Aged Care Facilities (TACF). This program draws on evidence from the development of Teaching Nursing Homes in the United States of America and Scandinavia, which have been shown to provide effective healthcare student training when they deliver a collaborative, inter-disciplinary learning environment where students can experience team-based care and opportunities for independent assessment [6,7].

The Wicking TACF Program aims to institute large-scale, aged care placements that build organisational capacity for evidence-based best practice and provide nursing students with a positive aged care experience to encourage them to seek employment in the sector. Quality clinical placements are important for supporting student nurses’ future employment choices [8], yet RACFs are unpopular as clinical training sites [9]. Central to the problem is a lack of RACF capacity to provide students with a positive placement experience [10]. The challenge for student nurse education in aged care is to make training clinically relevant when learners often work alongside less-qualified care staff. Previous studies have revealed that students spend 20% of their placement time with unregulated care workers, rising to 60% in some contexts [11]. Exploratory literature searches did not reveal any published studies that addressed interactions between care workers and undergraduate nursing students in the context of RACF clinical placements. However, North American research concerning graduate nurses’ first year in the aged care workforce indicated that care staff often take a lead role in facilitating adjustment to a complex work environment [12]. The literature that addresses student interactions with clinical mentors/preceptors in aged care usually focuses on engagements with qualified nurses [13,14]. When students engage with nursing staff, however, the experience can be either positive or negative. It has been reported that students value mentors’ expertise (as professional role models), but also identify a lack of capacity or desire to engage as clinical educators among some nursing staff [14,15]. When undertaking research into clinical placement experiences in aged care, some researchers have noted that a major difficulty with these programs is that there are seldom enough qualified, senior staff to provide effective mentorship for learners [16].

A small number of Australian studies have considered student experiences of care provision in the context of an RACF placement, although these studies do not address interactions with nursing or care staff. Three studies identified that negative student attitudes and lack of student engagement are common in aged care placements and that these are often associated with experiences of care provision [9,10,17]. A longitudinal study of nursing student attitudes to professional practice at the beginning and end of a degree program identified the aged care sector as the least preferred work environment [9]. A second study identified that learners often disregard RACF placements as being of low clinical relevance, particularly for the development of complex technical skills [10]. A more recent study reported that prevailing expectations for technical knowledge and challenge, where students have a collective identity of nursing as a hospital-centred profession, were rapidly extinguished during an RACF placement and replaced by a sense of disengagement associated with their perception of routinized patterns of care provision [17]. In this context, RACF staff reported that it became burdensome trying to include disengaged students in aged care work [17].

There are a number of potential explanations for nursing students’ lack of both engagement with and enthusiasm for aged care. A focus on technical challenges [10], as outlined above, cultural bias in nurse education that seeks alignment with medical and acute models of care [17], negative stereotypes of ageing [9], and media portrayals of nursing have arguably led to holistic and psychosocial models of care being viewed as inferior and unchallenging [5,18,19]. Feminist scholars argue while such care has symbolic value as a site for valorising (or revaluing) femininities such as intimacy, reciprocity, and relationships, within patriarchal value systems it is “easily discounted as a source of status or social worth” [20]. Since the early 19^th^ Century, caring within the practice of nursing has been regarded as a pillar of healing and comfort [21,22]. Bulfin states that nursing is caring theory realised and is underpinned by basic notions of concern, respect, and humaneness [22]. Of relevance to the present research, there is a notable dearth of evidence in the published literature that addresses hygiene care within the broader concept of caring. Personal hygiene care (hereafter referred to as hygiene care) includes such activities as toileting, bathing, changing clothes, hair and nail management, and dental care. Although there is evidence for specific elements of hygiene care – oral hygiene has received considerable attention in the literature [23] – there is relatively little information about how nurses engage more broadly with routine hygiene care and the clinical value of such activities for students. This study explores RACF care workers’ potential to develop strategies for teaching and learning that facilitate their capacity to engage nursing students’ interest in the performance of hygiene care activities, drawing on action research evidence.

## Methods

### Design

The study was conceived as critical action research. The TACF Program builds on a model of quality clinical aged care placements [24] and utilises an action research framework to support the capacity building processes employed in each RACF to enable staff to effectively mentor students. Action research is a collaborative approach wherein participants work together to reflect critically on their challenges with a view to understanding and improving their practices [25-27]. Core elements of action research include the involvement of participants as collaborators who identify an area of thematic concern that requires attention; the utilisation of spiralling steps of planning, action, reflection, and evaluation; and an overt focus on emancipation or change within a particular setting [28]. In the context of this research, an action research group was formed (referred to as the mentor group) to identify and critically reflect on the issues and work through them to take strategic action [27]. Action research group members worked through a research cycle that consisted of the identification of problems within the student placement context, planning and taking action to resolve problems, reflection and evaluation, and replanning as needed [27]. This study design has previously been successfully used by members of the project team to develop student placements [29], to build RACF staff capacity, centring on falls prevention [30,31], and to increase capacity among community nurses to facilitate chronic disease self-management [32]. In this study, one action research cycle involved mentor group members working with the first and third authors to develop teaching and learning strategies to engage nursing students’ interest in the delivery of hygiene care to RACF residents. Following an action research process allowed mentors to identify concerns with students’ perceptions of care provision, critically reflect on these issues, and create an action plan, which involved development of a new tool to assist carers to mentor students.

### Participants and setting

Participants were second year Bachelor of Nursing students (*n* = 10) who were randomly (by domicile) allocated a four-week (20 day) placement at a 140-bed RACF in May 2013 and their facility mentors (*n* = 17). All available participants involved in the action research cycle were included in the research. Nursing students were recruited at an information session in the weeks prior to the placement. Nursing and care worker staff were invited to volunteer to act as mentors and to form an action research mentor group, which first met in 2011.

### Data collection

Data for this study come from six action research group meetings held with the mentor group during a six-week period, including one prior to and one after the four-week nursing student placements, and from four feedback meetings (one per placement week) undertaken with the students. All meetings were undertaken by an experienced facilitator to ensure participants felt safe to honestly critique their experiences of the placement and were provided with an equal opportunity to contribute to the discussion. Mentors met in the first instance to discuss their plans for the placement and then, when the students arrived in the facility, to talk about their interactions and develop strategies for responding to emerging issues. Weekly student feedback meetings provided an opportunity for students to talk about their experiences of learning within the RACF. All meetings were audio-taped and transcribed verbatim.

### Ethical considerations

Informed, written consent was provided by all research participants prior to their involvement in the research. Involvement in the research was voluntary, although student participation in other placement activities was mandated as part of their university course requirements. The Human Research Ethics Committee (Tasmania) Network approved the project (H0011576).

### Data analysis

The analysis of qualitative data followed standard procedures for analysing social settings that have been reported in other studies of RACF clinical placements [33]. All meetings with students and mentors were transcribed to facilitate data analysis. Meeting transcript data were entered into a QSR International NVivo 10 database to facilitate organisation and coding. Descriptive coding (categorisation and ordering of all relevant data) and analytic coding (focussed and emergent induction) was performed to ensure that latent categories within the data were identified and that these were developed into thematic explanations of student and mentor experiences [34]. Transcribed data were coded by both the project officer and research assistant to increase the reliability of the analysis. The final analytical products were akin to thematic descriptions, including exemplary respondent quotations, which were referred to in this study as *case notes*.

### Rigour

In qualitative research, notions of rigour can be conceptualised as trustworthiness – the degree to which researchers can be confident that findings are credible, transferable, dependable, and confirmable [35]. Strategies to ensure sufficient rigour of qualitative research have been described in the nursing and methodological literature [36,37] and were used in the present research. These included the return of de-identified case notes to mentors at the beginning of weekly discussions. This served both to facilitate member checking [38] and to confirm the veracity of emerging themes [39] in order to develop a concurrent process of data collection and analysis.

A process of peer de-briefing between a project officer and research assistant was undertaken to check that both were satisfied with the main points of each discussion and that emerging analytic coding was supported. The research findings and emerging themes were also discussed with a team of researchers with extensive qualitative research experience and/or clinical expertise in the care of RACF residents. The prolonged engagement with two participant groups, six weeks with the mentors and four weeks with nursing students, as outlined above, also provided opportunities for checking and confirming the trustworthiness of the emerging analysis over time. The prolonged data collection ensured candid participant responses, through the development of a trusting relationship with the facilitator, as well as facilitating theoretical saturation of the data.

## Results

### Sample characteristics

Eight of the ten student participants were born in Australia. Mean age was 24 yrs (*SD* = 5). Only one student was male. The mentor group comprised 5 care workers and 12 nursing staff (enrolled or registered nurses). The mean age of care worker and nurse mentors was 41 yrs (*SD* = 16) and 47 yrs (*SD* = 15) respectively. All carer mentors had a certificate in the provision of care for older people. All mentors had worked in the facility for over one year and were female.

### Identification of the issues

The catalyst for the action research cycle was a series of negative encounters between student nurses and care workers reported in the weekly student feedback meetings and mentor action research meetings. Nursing students reported that they did not expect to engage with care workers during the execution of requisite placement activities and felt that it was inappropriate to participate in the provision of hygiene activities under their supervision. One student stated, “I feel like a carer rather than a nurse. I’ve just been doing cleaning and feeding [of residents]…whereas in hospital you do obs[ervations], you do other stuff and it makes you feel like a registered nurse” (BN2035, Wk1). Reinforcing this sentiment, another student spoke about her fears that participation in personal care activities, particularly hygiene care, undermined her nurse education and represented a lost opportunity:

If we all wanted to be carers we would come and work in a care environment. We’re all here to be registered nurses, we want to be doing [medication] rounds, we want to be giving eye drops…I understand that hygiene [care] and all of those sorts of things are very important, and they’re important whether you are in an acute setting or not, but there’s also a hell of a lot else that we would like to do (BN2040, Wk1).

Unsurprisingly, care workers reported feeling devalued as student mentors and felt their role in the RACF was undermined by students’ inability to understand the importance of hygiene care in the daily assessment of residents. During the second action research meeting in the first week of student placement, mentors expressed concern about the lack of respect students demonstrated when asked to participate in hygiene care and missed opportunities for student learning. Reflecting on their engagement with nursing students in the first week of placement, a carer mentor stated that the students displayed, a “lack of respect for our knowledge” (BMtr024, Wk3). The lack of perceived value in the personal hygiene task and the associated devaluing of carers was also evident to the nurse mentors: “[The students] couldn’t see that by providing hygiene care to [a resident] they’re assessing them – they couldn’t get that” (BMtrL006, Wk1). Students vocalised their concerns about the appropriateness of their involvement in the hygiene care to the nurse mentors, who became frustrated by their lack of engagement.

It was frustrating…you’d ask [students] how they’re going with their personal care and they weren’t impressed that they had to do that. There was still that attitude, “I’m a second year [nursing student], I’m doing my acute prac[ticum] next and I’m stuck in aged care” (BMtr023, Wk1).

From their initial placement experiences, mentors’ discussions highlighted the importance of working together to address problems in their interactions with students. Consistent with the action research process, during the second placement week, they were challenged by the facilitator to critically reflect on how they might foster nursing students’ learning so they developed a better understanding of the importance of hygiene care in RACFs and the relevance of this to their nurse education. In the context of these discussions, care worker mentors considered how their role added to the provision of care for residents and the operation of the RACF. Three themes emerged from this discussion: a) hygiene care provides an opportunity to assess residents, b) carers’ provide feedback to nursing on the basis of these assessments, and c) skills developed in the performance of hygiene activities are transferrable to acute care settings. As one carer recounted, provision of hygiene care facilitates the assessment of, “nose bleeds, prolapses, skin tears, skin redness, penis redness, splits in the groin, signs of constipation, redness behind the ears, or infections in the eyes or all sorts of things, spots and pressure areas” (BMtr021, Wk2). Furthermore, one carer noted, “We are the eyes and ears for the nurses – if we don’t do our job properly the nurses can’t [do their job]” (BMtr025, Wk2). Another argued, “In this environment we pass information on to our nursing staff. They don’t shower [the residents] so they wouldn’t know if there’s a pressure area unless there was some reason they needed to be looking at them” (BMtr024, Wk2). Making the link between resident assessment, provision of critical resident information to nursing staff, and the transferability of skills developed through the performance of resident hygiene, the care workers identified the importance of students having access to these experiences: “In the hospital environment, if someone needs a shower and can’t shower themselves, [nurses] don’t have carers so they need to know this stuff because they need to know what they’re looking for” (BMtr024, Wk2). Through these discussions, the mentors explicitly valued the key role played by care workers in the RACF’s inter-disciplinary health team.

### Action planning and taking action

Mentors articulated the value of the care worker role, the transferability of skills, and the importance of hygiene care as a form of assessment. Importantly, they also considered how these messages could be translated into opportunities for student learning during the placement. In an effort to operationalize these new understandings, the carer mentors suggested a tool be developed to assist students to identify the key assessment elements that are implicit in the provision of hygiene care. This tool became known as the Carer Assessment and Reporting Guide (the Guide). Reflecting on the necessity of the Guide, mentors spoke about the need to both inculcate respect for care workers and foster student engagement with important clinical information that is obtained during hygiene care. As one care worker mentor put it, the Guide “came from the lack of respect that we felt [the students displayed] for our knowledge and getting the students engaged into wanting to learn with us” (BMtr024, Wk3). Similarly another stated,

The students don’t even know that [care workers perform resident assessments], so creating the Guide was about making it explicit, so then you could talk to the [students] about it: “When we do hygiene [care], these are the things to look for” (BMtr022, Wk2).

The five carers within the mentor group subsequently worked together during the second and third placement weeks to create and implement the Guide. The guide provided prompts for the observation and assessment of resident care needs during the performance of hygiene care. It also connected these activities with core nursing competencies. The Guide addressed a range of assessment areas, including: a) cognitive ability (e.g. resident awareness of time, day, year); b) skin integrity (e.g. skin tears, pressure areas); c) hygiene (e.g. cleanliness of abdominal creases); d) bowels and urine (e.g. signs of constipation, incontinence, or blood); and e) mobility (e.g. review facility manual handling policy before engaging with residents). The Guide also outlined strategies for communicating more effectively with frail older people with dementia, reflecting care workers’ intimate knowledge of residents. Examples from the Guide include: a) ‘always enter the resident’s room with a quiet, calm, and happy disposition as your mood may affect the resident’; and b) ‘be mindful of maintaining residents’ independence and encourage them, as much as possible, to clean their own teeth (even if you need to assist) or wash their face’. During the third and fourth placement weeks the Guide was disseminated to all nursing students and care staff within the facility, including those outside the mentor group. Care worker mentors reported using the Guide as a cue to facilitate self-directed student learning and as a tool to support care workers evaluate student understanding.

### Evaluation and critical reflection

During the final feedback meeting, students reflected on their engagement with care staff and considered whether their original impressions of participation in hygiene care had changed. One student commented, “I feel like I’ve got better hygiene skills and I found [the Guide] useful because you have the same [residents you are looking after during the placement], so you can see changes in their skin and new things, and any deterioration, so that’s been good” (BN2037, Wk4). Another student reflected on the benefits of using the Guide in the presence of carers: “I was using [the Guide] with a carer when we were [providing resident care] and it listed pain and what we’d be looking out for, what’s causing the pain, why, and how long it’s been there” (BN2031, Wk4).

Mentors also reported changes in student attitudes during the final two placement weeks. They remarked on students’ growing appreciation of engagement with care staff and increased involvement in activities previously regarded as having low clinical value. For example, one carer noted,

One [student] was bored with [providing] hygiene [care], so I asked her to go and get [the Guide] out, and we went through it . . . She came up to me later as I was leaving and thanked me for a good morning, because I said to her, “I would like you to use this and challenge yourself against it and use [the Guide] as an assessment and then report to the nurse. Or go and read that client’s file and find out whether any of these things [in the Guide] are actually listed [in the file] – if they’re not, report it to the nurse and find out the process.” That made it more interesting for her (BMtr024, Wk4).

At the post-placement action research meeting, care worker mentors also critically reflected on their role in developing the Guide: “[We were] working out what we do and our job description, so [the Guide] captured that” (BMtr020, post-placement). They expressed surprise at the scope of their responsibilities and the implied skill sets as articulated in the Guide. One care worker recounted, “It’s interesting to see it all written down” (BMtr025, post-placement), while another commented, “We’re a lot more educated than we thought” (BMtr024, post-placement). As the discussions progressed, it was apparent the care workers had developed a new appreciation of their role in the inter-disciplinary environment of the RACF. One argued that developing the Guide had, “validated what we do, which gives us more confidence to say ‘this is how important [hygiene care] is’” (BMtr025, post-placement). Furthermore, the explicit acknowledgement of the scope of the care worker role also resulted in a perceived change in the way nursing staff collaborated with care workers:

Another carer and I actually had a bit of a giggle one afternoon because two registered nurses were sent in to assess a wound and they actually asked our opinions…They actually asked us “Do you think this needs a dressing?” That was nice (BMtr025, post-placement).

These findings suggest that developing the Guide not only supported student learning and carer mentorship, but also built self-esteem and acknowledgement among their nursing colleagues.

## Discussion

Research indicates that nursing students often struggle to engage with the learning opportunities in RACFs [9,17,40], although relatively few researchers have attempted to intervene to improve clinical placement experiences. Data from the present study are consistent with these findings and suggest that nursing students have negative attitudes towards aspects of the aged care placement when they first enter an RACF. Nursing students were initially dissatisfied with being asked to participate in the provision of resident hygiene with care workers. This engagement was viewed as an opportunity cost that took students away from what they perceived as more valued training experiences accessible in acute care settings. Aged care was regarded as a professional backwater incongruent with contemporary nurse training. This is unsurprising given evidence which highlights an increasing tendency for nurses to seek validation of their professional competence through the development of complex technical skills that are closely aligned to a medical model of health within acute care settings [19,41]. It is possible that nursing students’ emerging professional identity, one that arguably privileges technical competence in hospital settings and modelling from registered nurses, is threatened when they are confronted with the apparently low-skilled work of providing hygiene care to frail older people.

While initially frustrated with students’ negative attitudes at the outset of the placement, mentor group members critically reflected on emerging challenges and developed new understandings of their situation as part of an action research process [42]. Working through a sequential process of issue identification, planning, action taking, and critical reflection, mentors recognised that students were situating care workers as hierarchically inferior and thereby diminishing their worth within the aged care sector and the educational placement context. This is consistent with evidence in the literature, which suggests that care workers’ role in aged care is largely unacknowledged or poorly regarded [43]. A lack of acknowledgement and respect has been identified as a mediating factor in job dissatisfaction and staff turnover among this cohort [43,44]. Despite negative perceptions about their role, studies of care worker engagement have shown they develop intimate relationships with residents, take pride in the care they provide, and seek to establish long-term careers within the aged care sector [44]. The mismatch between student perceptions of the role and carer experiences and competency may contribute to an explanation for care workers’ indignation at the perceived disrespect that they were shown. Through critically reflecting on their challenges, mentors developed a new appreciation of students’ concerns regarding the strong imperative to develop competence for a future role as a nurse, which has been well-documented in the literature [41]. During the action research meetings, carer mentors were challenged to facilitate student learning in ways that would help them to appreciate the value of perceived low-status care work in assessing and identifying residents’ ongoing care needs.

In developing the Carer Assessment and Reporting Guide, carer mentors worked to explicitly acknowledge the complex nature of their role for both students and their nursing colleagues. The findings of this project suggest that in the context of their involvement in an action research process, such changes in understanding challenged traditional hierarchical relationships and set up more collegial relations between nurses and their care worker colleagues. For the students, the Guide systematically facilitated exposure to the complexity implicit in the delivery of hygiene care. Student accounts indicate that they developed a new appreciation of the value of this work and the importance in making ongoing assessments of residents’ care needs. This might be seen as care workers supporting students to reconnect with the care imperative that has underpinned nursing practice since Florence Nightingale attended to dying and convalescing Crimean War soldiers [21]. Yet the schism between nursing student expectations of their professional role and what they encounter in RACFs arguably reflects a broader tension within the health sector between hospital and community care.

Recent decades have seen the aged care sector move away from an overemphasis on safety, uniformity, and medical issues, towards person-centred care, individual choice, and quality of life [45]. A key driver for this cultural change in RACFs has been the development of more respectful, person-centred interactions between residents and care staff [45], in line with arguments that care revalues relationships and intimacy [20]. The growing focus on technical skills and specialisation in nursing generally is not only at odds with cultural change in RACFs, but is also incongruous with demographic and health sector shifts that will see manifold increases in the cohort by the middle of the century and attendant rises in prevalence of non-communicable health conditions, including dementia [46]. By reconnecting with the care imperative, students arguably develop core nursing skills that will help them to better interact with frail older people and become better prepared for 21^st^ Century healthcare.

The findings of the present study suggest that care workers can provide effective mentorship to undergraduate nursing students, but that efforts and resources are required to engage learners with the clinical value of care work. This emphasises the importance of giving aged care staff, particularly those engaged in mentoring nursing students, the opportunity to meet and critically reflect on the challenges and opportunities within this educational framework. This supports evidence from international studies, which indicates that Teaching Nursing Homes provide effective nurse training in the context of a collaborative, inter-disciplinary learning environment, where students can experience and contribute to team-based care [6,7].

Due to the small size of the student and mentor cohorts, further research is required to confirm the findings. The addition of larger-scale quantitative measures of nursing students’ pre- and post-placement attitudes towards care staff and hygiene tasks would provide useful triangulation. Strengths of the study include longitudinal researcher engagement with participants and the opportunities for carers to reflect critically on ways to improve student learning experiences and attitudes as part of an action research cycle. Repeated discussions with students and mentors over six weeks provided opportunities for member checking the emerging analysis as the placement progressed. The action research process provided an effective mechanism for problems to be identified by the mentor group, strategies for action to be discussed and implemented, and both groups to reflect on changing attitudes towards care provision.

## Conclusion

It is concerning that nursing students enter a four-week RACF clinical placement with negative attitudes towards both provision of resident care and interactions with care workers. This suggests that more emphasis is required in nurse education to instil an appreciation for the importance of direct patient interaction and communication with care staff, as well as recognition of complex health challenges and subtle changes in condition that confront health professionals during resident interaction. Through implementation of the Guide during an action research process, nursing students learned to value intimate resident care and inter-professional teamwork, while carers developed a renewed sense of their own knowledge and contribution within the RACF.

## Abbreviations

RACF: Residential aged care facility
TACF: Teaching aged care facility
